# Formation of stable Si–O–C submonolayers on hydrogen-terminated silicon(111) under low-temperature conditions

**DOI:** 10.3762/bjnano.6.3

**Published:** 2015-01-05

**Authors:** Yit Lung Khung, Siti Hawa Ngalim, Andrea Scaccabarozzi, Dario Narducci

**Affiliations:** 1 University of Milan-Bicocca, Department of Materials Science, Via R. Cozzi 53, I-20125 Milan, Italy; 2 Regenerative Medicine Cluster, Advanced Medical and Dental Institute (AMDI), Universiti Sains Malaysia, Penang, Malaysia

**Keywords:** hydrogen abstraction, thermal hydrosilylation, UV-initated hydrosilylation, X-ray photoelectron spectroscopy

## Abstract

In this letter, we report results of a hydrosilylation carried out on bifunctional molecules by using two different approaches, namely through thermal treatment and photochemical treatment through UV irradiation. Previously, our group also demonstrated that in a mixed alkyne/alcohol solution, surface coupling is biased towards the formation of Si–O–C linkages instead of Si–C linkages, thus indirectly supporting the kinetic model of hydrogen abstraction from the Si–H surface (Khung, Y. L. et al. *Chem. – Eur. J.* **2014,** *20,* 15151–15158). To further examine the probability of this kinetic model we compare the results from reactions with bifunctional alkynes carried out under thermal treatment (<130 °C) and under UV irradiation, respectively. X-ray photoelectron spectroscopy and contact angle measurements showed that under thermal conditions, the Si–H surface predominately reacts to form Si–O–C bonds from ethynylbenzyl alcohol solution while the UV photochemical route ensures that the alcohol-based alkyne may also form Si–C bonds, thus producing a monolayer of mixed linkages. The results suggested the importance of surface radicals as well as the type of terminal group as being essential towards directing the nature of surface linkage.

## Introduction

Forming covalently-attached organic submonolayers on silicon remains one of the challenges in surface science. In order to gain access to the electronic properties of silicon, it is imperative that the organic layer on the top surface be kept thin enough to avoid a masking of the intrinsic properties of silicon, especially in biosensing application [1]. So far, hydrosilylation is among the most commonly accepted techniques to graft organics onto silicon surfaces [2–6]. It is the process during which unsaturated carbon reacts with hydrogen-terminated silicon (SiH) to form a stable submonolayer through covalent Si–C linkages at the surface. The reaction can typically be mediated through catalysts or Lewis acids [3–4], through an intermediate halogenation followed by Grignard chemistry [7], through UV irradiation on the surface [8] or thermally driven [9–10]. In recent years, thermal hydrosilylation has emerged as an attractive alternative due to the lack of potentially contaminating catalysts as well as the low process costs. The general consensus on the mechanism of hydrosilylation of the bulk silicon surface proposed by Linford et al. suggests a self-propagating chain mechanism that ultimately leads to densely packed layers. It is considered to be a self-repeating three-step reaction [11] after the initial radicalization of the silicon surface:

[1]
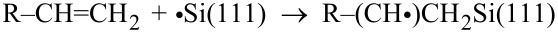


[2]
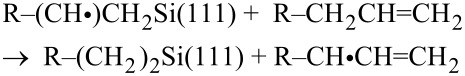


[3]
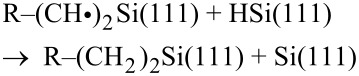


The conditions by which Linford et al. performed the reaction were very stringent and regardless of variations in the experimental approach in later studies by other authors, the basis of a silyl radical reacting with unsaturated C–C bonds remained undisputed. However, as early as 2005, Wood et al. brought to attention that the cleavage of Si–H to form initial silyl radicals might not be the only mode for hydrosilylation to occur [12]. Typically, the commonly accepted notion is that thermal hydrosilylation requires temperatures above 150 °C in order to cleave the silicon–hydrogen bond at the surface to form surface radicals. However previous studies had shown that hydrosilylation could also proceed at a lower temperature (110 °C). Wood et al. further suggested a reaction mechanism in which trace oxygen is involved in the extraction of hydrogen off from the hydrogenated silicon surface.

One question to address would be the actual reaction preference of the Si–H surface when exposed to both an alkyne and an alcohol at lower temperatures, i.e., whether the surface would still undergo hydrogen abstraction in the presence of a competing reactant. One of the classical competitor to alkynes forming Si–C on Si–H are alcohols. They were previously reported to react with Si–H to form stable Si–O–C linkages [13]. To examine this point, we proposed a comparative study between two modes of hydrosilylation (thermal and UV photochemical) for a bifunctional alkyne. Alkynes were deliberately chosen due to their higher reactivity towards hydrogen-terminated silicon compared to alkenes. The main theme of this study is to examine whether hydrogen extraction is a probable mechanism for surface reaction at low temperatures. Two alkynes were selected, namely 4-ethynyl-α,α,α-trifluorotoluene (trifluoroalkyne), whose trifluoride functional group serves both as both a surface marker (in the C 1s reaction) and as a means of raising the surface hydrophobicity upon functionalization of the alkyne, and 4-ethynylbenzyl alcohol (ethynylbenzyl alcohol) whose hydroxy (OH) group may initiate a nucleophilic attack on the Si–H surface [9] while the alkyne termination can present itself for reaction to the same surface through hydrogen abstraction [12,14]. The hypothesis is that, for thermal hydrosilylation, at low temperature, two mechanisms may potentially occur to form two different linkage (Si–O–C and Si–C) [12,14], namely hydrogen abstraction through trace oxygen or a nucleophilic attack on the silicon surface for the ethynylbenzyl alcohol. On the other hand, if the surface is activated through UV irradiation, both mechanisms can be deemed to be unneccesary, thus facilitating the grafting reaction of the molecule through the alkyne end, in turn, forming a stable Si-C linkage. On the other hand, a trifluoroalkyne was also selected to demonstrate the viability of the hydrogen abstraction model by observing the nature of the linkage formed considering that this molecule could only react at the alkyne end. Thus, the eventual presence of Si–O–C and Si–C from surface analysis in our controlled setup would give impetus towards the acceptance of the hydrogen abstraction model for low-temperature hydrosilylation. The role of oxygen in the low-temperature hydrosilylation reaction can then be better understood from this experimental approach.

## Result and Discussion

**Figure 1:**
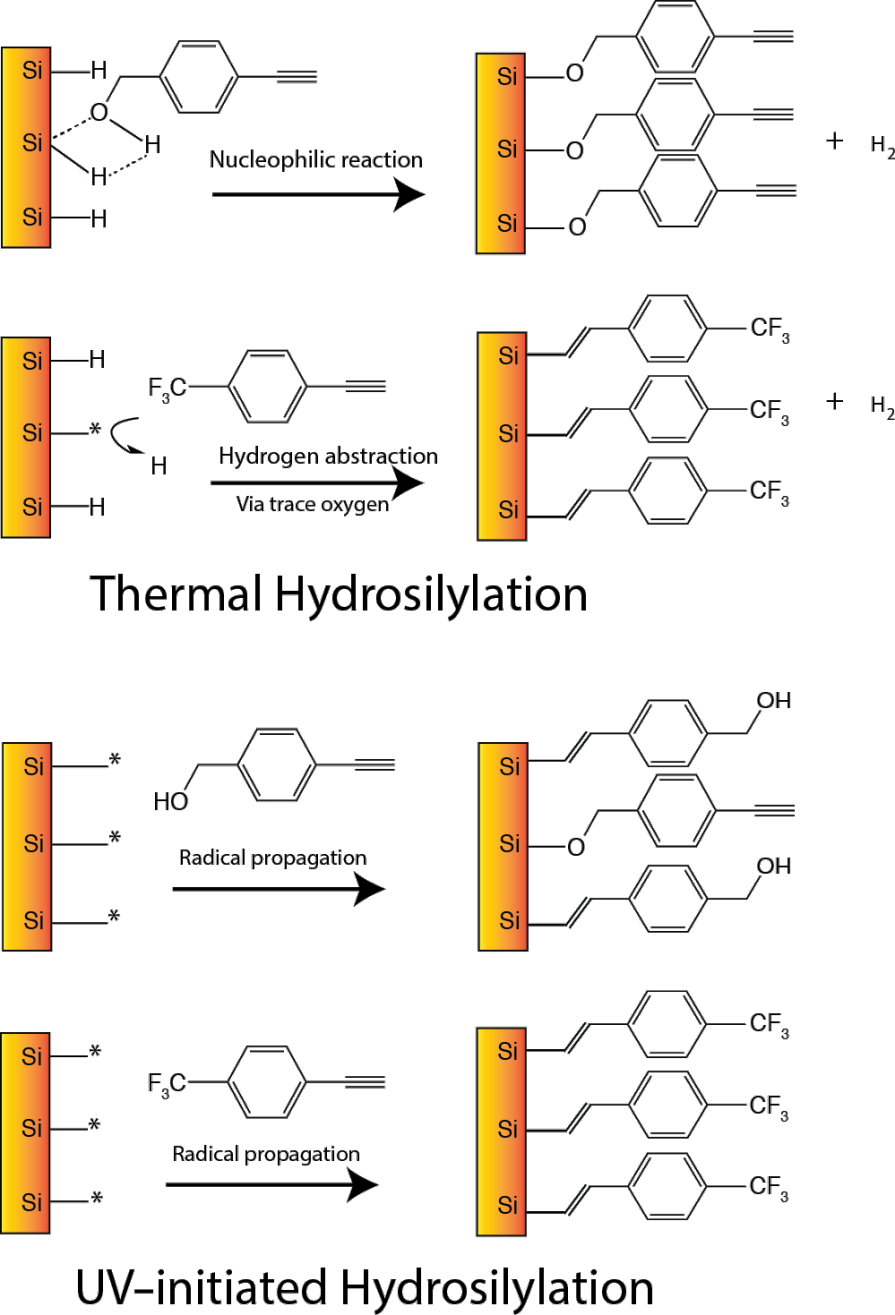
Hypothetical reaction pathways of ethynylbenzyl alcohol and trifluroalkyne during thermal and UV-initiated hydrosilyation.

To help understand the role of oxygen during hydrosilylation, a direct comparison of the reactivity between both thermal and UV-initiated hydrosilylation was made for two different alkyne species. Trifluoroalkyne was employed to demonstrate the formation of Si–C linkages through hydrogen abstraction by trace oxygen and the level of oxygen was greatly reduced by a series of degassing steps similar to those described by Ciampi et al. [15]. On the other hand, ethynylbenzyl alcohol was used to react with the surface via a nucleophilic route from the hydrogen-terminated surface during low-temperature hydrosilylation. It is envisaged that at low temperatures (<150 **°**C), the Si–C bonds at the surface are not cleaved to form radicals. Thus, in order for surface grafting to form Si–C linkages, it is necessary for the hydrogen to be abstracted from the surface via oxygen species present in solution (Figure 1). In our deliberate thermal setup, there is also the possibility of grafting via Si–O–C linkages instead of the nominal Si–C linkages typically associated with thermal hydrosilylation. However, we envisage that under UV irradiation, the surface would be pre-activated to form silyl radicals and surface grafting could proceed to form both the Si–O–C and the Si–C as Hacker et al. had previously demonstrated the viability of creating thin Si–O–C films with saturated alcohol through UV irradiation [13]. Such observation would further reinforce hydrogen abstraction as a viable mechanism for low temperature hydrosilylation. It is important to note that the film produced on the silicon surface can only described as a sub-monolayer as attaining a full surface coverage in which every silicon atom is occupied would be technically impossible due to steric hindrances and this has been well discussed in literature [12,16].

**Figure 2:**
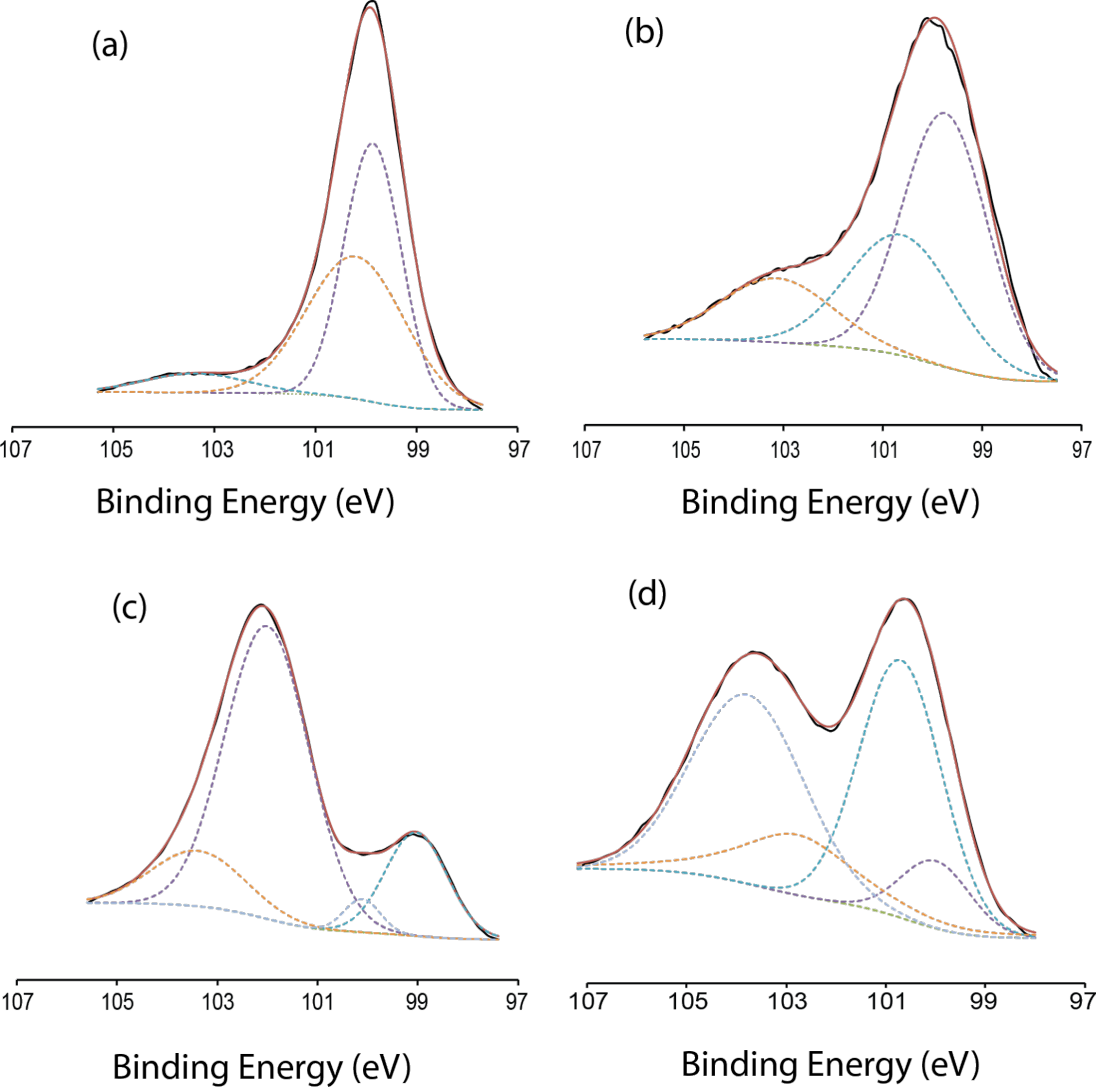
High-resolution XPS Si 2p spectra of the surface (a) thermally functionalized with trifluoroalkyne, (b) functionalized with trifluoroalkyne through UV-irradiation, (c) thermally functionalized with ethynylbenzyl alcohol and (d) functionalized with ethynylbenzyl alcohol though UV-irradiation.

High-resolution XPS analyses were performed on thermally grafted as well as UV-irradiated surfaces well for both trifluoroalkyne and ethynylbenzyl alcohol (Figure 2). The Si 2p spectra for thermally treated trifluoroalkyne and ethynylbenzyl alcohol exhibited the characteristic peaks (Si 2p_3/2_ (99.7–99.9 eV) and Si 2p_1/2_ (100.2–100.7 eV)) for elemental silicon while the broad distribution at 103.5–104.2 eV was attributed to the various Si–O*_x_* species [17–18]. Interestingly, for the trifluoroalkyne samples, the oxidation (Si–O*_x_*) was observed to be higher for the UV-irradiated surfaces (Figure 2b) as compared to that from thermal hydrosilylation (Figure 2a). This could be interpreted as a consequence of the higher concentration of surface radicals under UV exposure that rendered the surface more susceptible towards oxidation from residual oxygen (O_2_) in solution. As the temperature of the thermal hydrosilylation setup was less than 150 **°**C, there were no radicals formed at the silicon surface that was thus more stable towards oxidation during the reaction time. XPS analysis on the ethynylbenzyl alcohol revealed that for thermal hydrosilylation, the elemental Si peak intensities were reduced (99.6 eV and 100.1 eV) while an intense peak at 102.1 eV was observed in turn. Considering its position, it was unlikely to be oxide (≈103.7 eV) and previous reports had reported this position to be a strong indicator for the Si–O–C linkage. This is the first evidence that surface grafting of ethynylbenzyl alcohol had occurred through the Si–O–C linkage instead of the Si–C linkage.

When the surface underwent UV irradiation (Figure 2d), the level of oxidation (103.7–104.7 eV) increased significantly. The broad peak centered at 102.5 eV was attributed to Si–O–C [19–20]. The intensity of the elemental Si (99.8 eV and 100.3 eV) had also increased in comparison to that of the thermal hydrosilylation samples (Figure 2c). Coincidentally, Si–C was normally observed in the literature at 100.2–100.4 eV [21–22] and often overlapped with the Si 2p_3/2_ and Si 2p_1/2_ signatures. Therefore the observed increment at the 100.0–100.4 eV in conjunction with assignment at 102.5 eV could be taken as an indication that the UV-initiated grafting of ethynylbenzyl alcohol yielded two linkages, Si–C and Si–O–C. The increase in oxidation as highlighted by the broad peak at 103.7–104.7 eV may also be explained by the UV-initiated surfaces being more susceptible towards oxidation.

**Figure 3:**
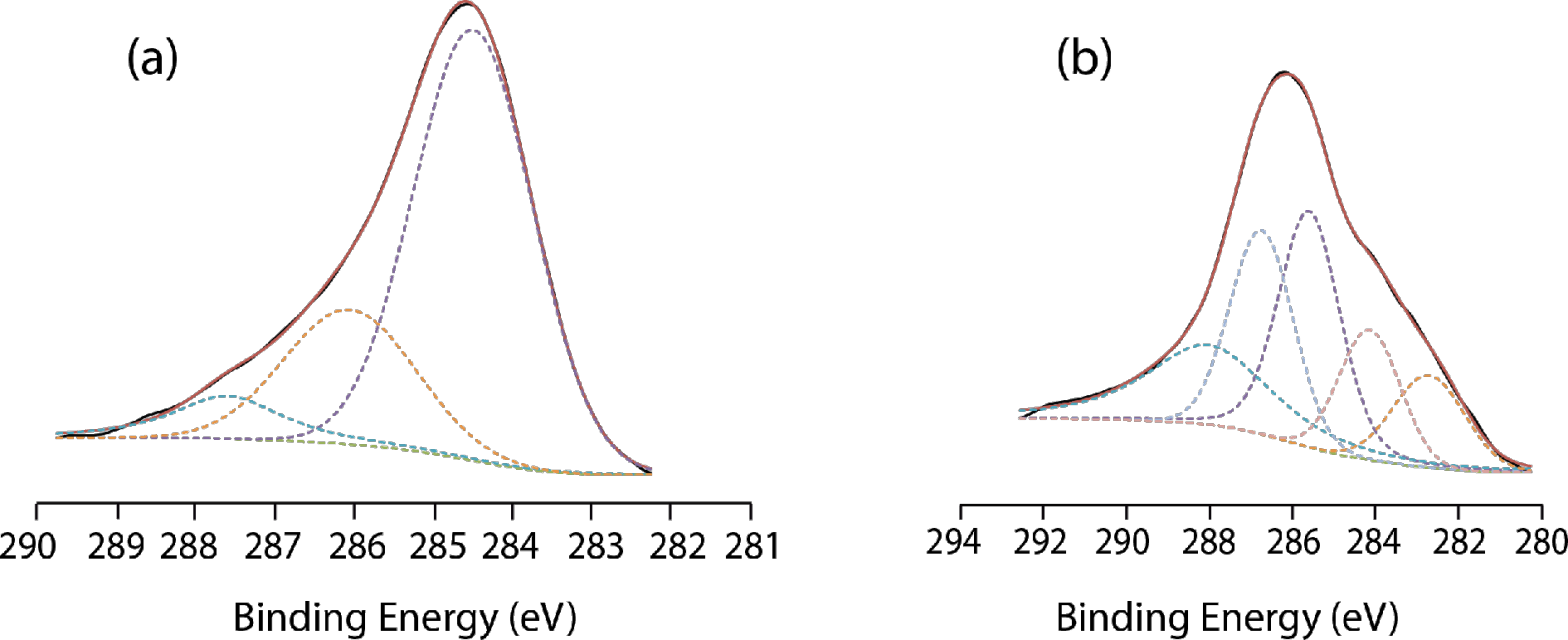
High-resolution XPS C 1s spectra of surfaces (a) thermally functionalized with ethynylbenzyl alcohol and (b) functionalized with ethynylbenzyl alcohol through UV initiation.

To study the nucleophilic reaction that gave rise to the Si–O–C linkage, high-resolution C 1s spectra were taken after both the thermal and UV-initiated hydrosilylation of ethynylbenzyl alcohol. The C 1s scan of the trifluoroalkyne would not serve any purpose considering the end product would be a Si–C linkage and cannot be used to examine the nucleophilic reactions as mentioned. Thus, as shown in Figure 3a, upon thermal grafting, a broad peak centering at 284.6 eV was attributed to that of sp^2^ C–C (as evident from aromaticity of the ethynylbenzyl alcohol) as well as adventitious C–C from the exposure of the surface to air [23–25]. Interestingly, the peak at 286.3 eV could be attributed to the epoxy type linkage (C–O–R) as previously reported in literature [26]. In view of the possible Si–O–C linkage at the surface, this assignment was deemed as representation of this linkage. The broad peak at 287.7 eV could be assigned to π–π carbon satellites, possibly arising from the aromatic stacking of the benzyl rings [27]. On the other hand, the high-resolution C 1s spectra of the UV-initiated functionalization with ethynylbenzyl alcohol yielded several peaks suggesting the presence of both Si–C and Si–O–C. While the peaks at 284.2 eV and 285.3 eV could be attributed to sp^2^ C–C and adventitious C–C, the most important peak assignment belongs to the signal centered at 282.6 eV [23,28–29], which gave the strongest evidence for Si–C linkages [30]. On the other hand, the peak at 286.3 eV proposed the presence of Si–O–C linkages on the same surface. Nonetheless, the Si 2p spectra suggested that under UV irradiation of ethynylbenzyl alcohol, due to the reactivity of the surface, an OH-terminated alkyne might react from both ends to the surface. Furthermore, by measuring the area under the peaks after a Shirley background subtraction and an automatically assigned Gaussian–Lorentzian fit with the XPS peaks software for both Si–O–C and the Si–C peaks, we found that the surface had been decorated at a Si–O–C (286.3) ratio of 2:1 relative to Si–C (282.6 eV). Hence, only a third of the surface had been functionalized through Si–C linkages.

**Figure 4:**
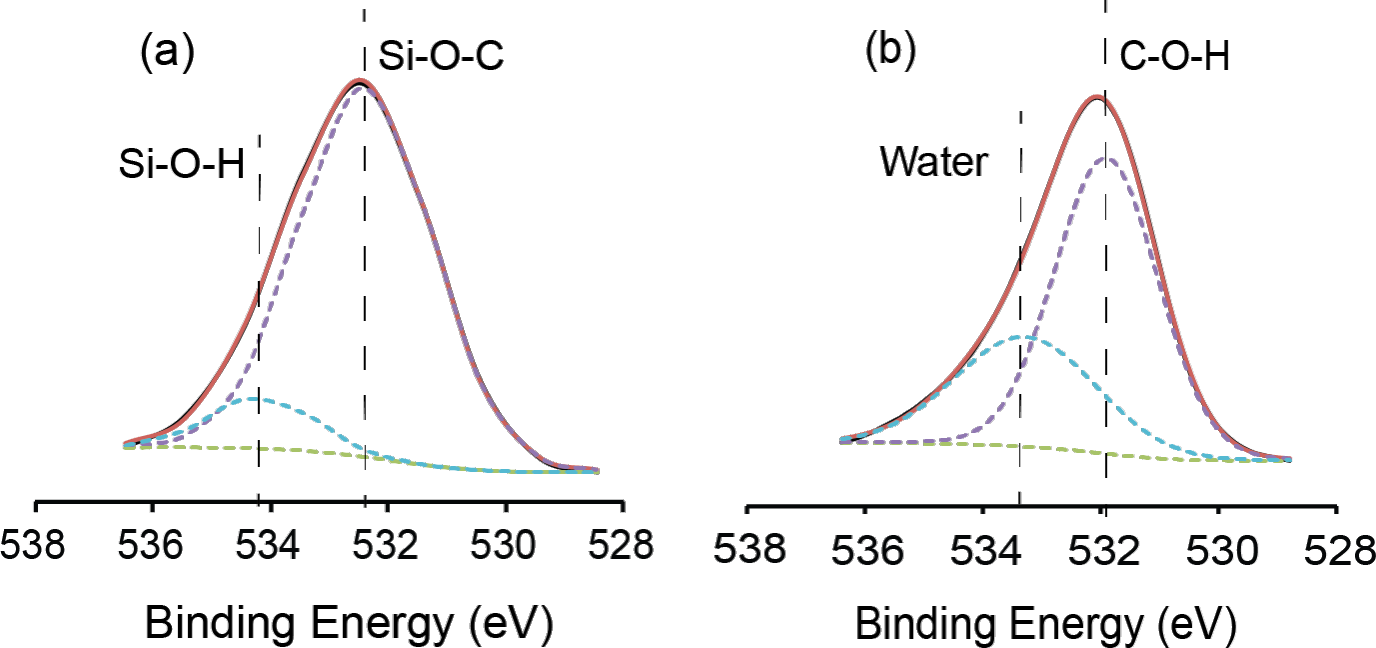
High-resolution XPS O 1s spectra of surfaces (a) thermally functionalized with ethynylbenzyl alcohol and (b) functionalized with ethynylbenzyl alcohol through UV irradiation.

High-resolution O1s spectra also helped to explain further the nature of the oxide on the surface, whether the oxide was inherent to the silicon surface or whether the oxide is bound to carbon or silicon as in the Si–O–C linkage. As shown in Figure 4a, on the thermally functionalized surface for the ethynylbenzyl alcohol, the main O 1s peak was observed centered at 531.9 eV. The main peak of the UV-functionalized surface (Figure 4b) was positioned at 532.4 eV. This peak can easily be assigned to the characteristic C–OH bond [31–33] of the hydroxy end groups of the ethynylbenzyl alcohol molecules that were linked to the surface through the alkyne end. The upshift of 0.4 eV for the main O 1s peaks between the two different reactions suggested that the OH-group of the ethynylbenzyl alcohol has different environments with respect to the surface. During thermal hydrosilylation, the lower binding energy can only suggest that OH had been cleaved to form a linkage to the surface as previously reported by Shao et al. [34]. Although in both of the mentioned linkages (Si–O–C and C–OH) in this paper, the carbon atoms are technically sp^3^ hybridized. Yet, the environment of the bond is considered to be very different. Compared to the exposed C–OH group of the ethynylbenzyl alcohol molecule bound to the surface through the alkyne group, an oxygen atom in the Si–O–C linkage would experience a difference in electronegativity (silicon is marginally less electronegative compared to hydrogen). The arrangement of an oxygen atom sandwiched between a silicon and a carbon atom would result in an increase in overall electrostatic repulsion and this will subsequently decrease the bonding energy, as was reported previously in literature [35]. From the O 1s spectra, we were able to observe this reduction in binding energy of O 1s in the thermal hydrosilylation samples and thus concluded that the predominant oxygen species in these samples had been associated with the Si–O–C linkage while those produced from UV-initiated hydrosilylation were C–OH. The secondary peak for the thermal hydrosilylation (Figure 4a) at 533.3 eV was indicative of SiO_2_ from oxidation [36] while the secondary peak for the UV-initiated hydrosilylation (Figure 4b) was centered at 534.3 eV, which was nominally linked to absorbed water on the surface [37]. What was interesting was that despite the wide FWHM for both samples (2.81 for thermal hydrosilylation and 2.07 for UV-irradiated surfaces), the absorbance of the water peak was only observable for the UV-irradiated surfaces. Considering that the exposed end groups on the surface of the UV-irradiated samples were C–OH, the reason for the absorbance of water is considered to be a more hydrophilic surface.

To further examine the nature of the grafting, contact angle (CA) measurements were performed and the results were are shown in Table 1. The values for the trifluoroalkyne, both after thermal and UV-initiated hydrosilylation, were very similar, namely (84.0 ± 1.5)**°** and (83.5 ± 0.5)° (Table 1). But this was not the case for the ethynylbenzyl alcohol. After the thermal hydrosilylation, the CA value was 89.6 ± 3.0**°**, higher than for the CF_3_-terminated trifluoroalkyne. One would imagine that the trifluoroalkyne would exhibit a higher hydrophobicity due to its fluoro-group termination while the higher values observed for the ethynylbenzyl alcohol could only be explained by the formation of Si–O–C bonds, with the free alkyne group exposed from the surface. On the other hand, upon UV irradiation of the surface, the CA values for the ethynylbenzyl alcohol were clearly reduced to (67.4 ± 4.1)°. Together with the XPS C 1s and O 1s high-resolution spectra obtained on these surfaces, the only sensible explanation for this was that both Si–O–C and Si–C linkages were formed on the surface, thus creating a patchy surface with an intimate mixture of moieties exposing hydroxy or alkyne groups. This would certainly reduce the surface wettability as reported in previous reports on heterogeneous monolayer-like assemblies on surfaces [38–39]. What was also interesting was that the atomic concentration, listed in Table 1, had revealed that the level of oxidation (O 1s) was significantly higher at UV-irradiated surfaces for trifluoroalkyne compared to the thermally treated surfaces and one possible explanation was that the UV-initiated hydrosilylation was carried out at room temperature while thermally treated surfaces were performed at 130 °C which would likely exclude water from the reaction system. This may allow for more extensively oxidation to occur since the radicalized surface was highly susceptible to oxidation. The O 1s spectra from the ethynylbenzyl alcohol had been already discussed in the previous section with C–O contributing to the high percentage of oxygen observed.

**Table 1:** Sessile droplet contact angle measurements of the two surfaces hydrosilylated with the two alkynes. The atomic concentration (atom %) from XPS survey spectra is also as listed below for the two different reaction mechanisms.

contact angle				

	thermal hydrosilylation		UV-inititated hydrosilylation	
	
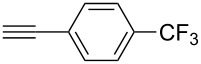	(84.0 ± 1.5)°		(83.5 ± 0.5)°	
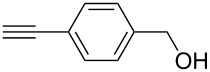	(89.6 ± 3.0)°		(67.4 ± 4.1)°	

atomic concentration (atom %) after thermal hydrosilylation				

	C 1s	Si 2p	O 1s	F 1s
	
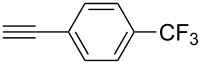	14.63	66.18	15.21	3.97
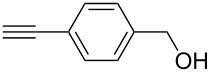	68.32	15.40	16.19	—

atomic concentration (atom %) after UV-initiated hydrosilylation				

	C 1s	Si 2p	O 1s	F 1s
	
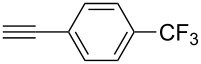	14.21	50.39	32.18	3.22
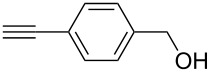	19.13	34.72	48.15	—

## Conclusion

From the XPS analysis and the contact angle measurements, several conclusions can be drawn from this study. Firstly, the efficacy of the low-temperature thermal hydrosilylation was heavily dependent on the presence of oxygen species. In the absence of oxygen carrying organics, it was possible that trifluoroalkyne was grafted through Si–C linkages because of trace amounts of oxygen. Despite the thorough degassing method used, it was not possible to remove all oxygen in a non-vacuum environment. Secondly, it was noticed that the UV-irradiation had created a highly reactive surface that was reacted with both OH-terminations and alkyne-terminations of the molecules. In the thermal setup for the ethynylbenzyl alcohol, a strong predominance of Si–O–C was observed. This suggested that the role of hydrogen abstraction from the surface through residual oxygen in the thermal setup is minimal as the absence of Si–C bonds from XPS also indicated that the reaction only yielded Si–O–C linkages despite the long reaction time of 18 h. The results reported here shed light on the issues related to OH reactivity at low temperatures as well as on the indiscriminate reactivity of the Si radicals formed at the surface. This information is important with regard to the hydrosilylation of OH bearing species to a silicon surface.

## Experimental

### Materials

Silicon wafers (111), were boron-doped (resistivity of 0.01–0.018 Ω·cm) and were used in this experiment. Sulfuric acid (Aldrich) and hydrogen peroxide (BDH Prolabo) were of semiconductor grade. 4-ethynylbenzyl alcohol and 4-ethynyl-α,α,α-trifluorotoluene were purchased from Sigma-Aldrich. All other chemicals, unless stated otherwise were used as received without further purification.

### Thermal reaction protocol

Similar to the methodology as described by Ciampi et al [15], silicon wafers approximately 20 × 20 mm^2^ were cleaned for 30 min in hot Piranha solution (95 **°**C, hydrogen peroxide (33%)/conc. sulfuric acid, 1:3 (v/v)). The samples were then submerged in a solution of 2.5% hydrofluoric acid for 1.5 min. Subsequently, the samples were placed to a degassed (through a minimum of 20 freeze-pump-thaw cycles) solution of 4-ethynyl-α,α,α-trifluorotoluene (0.3 M in mesitylene). The sample was kept under a stream of nitrogen while the reaction vessel was immersed in an oil bath set to 130 **°**C for 18 h. After the reaction, the flask was carefully opened and the functionalized surface samples were exposed to the atmosphere and subsequently rinsed and sonicated in copious amounts of chloroform, ethyl acetate, and then ethanol before being analyzed.

For the 4-ethynylbenzyl alcohol-based layer, the silicon surface was also functionalized in similar fashion and with the same molar concentrations. The functionalized surface samples were rinsed consecutively with copious amounts of chloroform, ethyl acetate, and then ethanol before being analyzed.

### UV-initiated hydrosilylation

Silicon wafers were pre-prepared in similar fashion to that in the thermal hydrosilylation protocol. Subsequently, the surfaces were transferred, taking care to completely exclude air from in the reaction vessel (a custom-made fused silica flask), to a degassed (through a minimum of 20 freeze-pump-thaw cycles) sample of 4-ethynyl-α,α,α-trifluorotoluene (0.3 M in mesitylene). The surface was irradiated with 254 nm (4.88 eV) UV radiation was provided by a commercial 6 W Hg tube. Any shorter wavelength component from the lamp (typically the 185 nm line) was filtered out by using a coloured glass filter with a transmittance of lower than 1% outside the 220–400 nm band. The choice of custom-made quartz Schlenk flask made of fused silica ensures a very high transmittance of the 254 nm light to the sample, up to 90%.

The experimental setup is arranged with the UV lamp held in vertical position with an adjustable distance from the reaction vessel, so that the UV light impinges perpendicularly to the sample surface and the power density can be easily varied. Vessel and lamp are enclosed in a dark box so that no light other than that from the UV lamp can reach the sample.

A calibration of the light intensity was performed by using a large area calibrated silicon photodiode from Hamamatsu photonics, showing that the experimental setup is capable to deliver 254 nm UV light intensities from 1.2 mW/cm^2^ down to 100 μW/cm^2^ (lower values can be easily obtained by inserting other filters). The lamp-to-sample distance was adjusted in order to have a power density of 700 μW/cm^2^. The surfaces were exposed to the UV irradiation for 2 h, then rinsed consecutively with copious amounts of chloroform, ethyl acetate, and then ethanol before being analyzed.

### Contact angle measurements

The water contact angle (CA) values were acquired on a Dataphysics OCA-20 goniometer setup at room temperature in ambient atmosphere. This instrument consists of a CCD video camera with a resolution of 768 × 576 pixels that can take up to 50 images per second. For each sessile droplet measurement three separate 5 μL droplets were dispensed onto the selected sample and the drop images were recorded. All drop images were then processed by an image analyzer that calculated both the left and right contact angles from the droplet shape with an accuracy of ±0.1°.

### X-ray photoelectron spectroscopy (XPS)

The XPS wide scan spectra were acquired by using an AXIS Ultra DLD, Kratos, equipped with an Al Kα X-ray source (1486.6 eV) at 10 mA, 15 kV, analyzing a 300 × 700 µm area under ultra-high vaccum (3.9·10^−9^ Torr). Analyses were performed in the hybrid lens mode with the slot aperture and the pass energy of the hemispherical analyzer set to 100 eV for the survey scan. High-resolution spectra were obtained for the C 1s, F 1s, Si 2p and O 1s energies for all samples. The spectra were subsequently analyzed by using the built-in Kratos Vision 1.5 software.
